# Dust Weight and Asthma Prevalence in the National Survey of Lead and Allergens in Housing (NSLAH)

**DOI:** 10.1289/ehp.9412

**Published:** 2006-11-07

**Authors:** Leslie Elliott, Samuel J. Arbes, Eric S. Harvey, Robert C. Lee, Päivi M. Salo, Richard D. Cohn, Stephanie J. London, Darryl C. Zeldin

**Affiliations:** 1 Laboratory of Respiratory Biology, Division of Intramural Research, National Institute of Environmental Health Sciences, National Institutes of Health, Department of Health and Human Services, Research Triangle Park, North Carolina, USA; 2 Constella Group, LLC, Durham, North Carolina, USA

**Keywords:** allergens, cross-sectional, environmental, house dust, respiratory

## Abstract

**Background:**

Settled dust has been used in studies to assess exposures to allergens and other biologically active components, but it has not been considered in the aggregate in relation to respiratory health outcomes in the general population.

**Objective:**

We addressed whether total house dust weight, an index of total dust exposure, was associated with respiratory health outcomes in the National Survey of Lead and Allergens in Housing (1998–1999) (NSLAH).

**Methods:**

NSLAH was a cross-sectional survey designed to represent permanently occupied housing units in the United States. In each household, a questionnaire was administered and settled dust was vacuumed from five locations. Linear regression models were used to identify predictors of dust weight; logistic regression models were used to examine the relationship between dust weight and asthma and wheeze.

**Results:**

Dust weight samples were available for 829 households, and survey information was available for 2,456 participants (children and adults). Lower income, older homes, household pets, having a smoker in the house, and less frequent cleaning predicted higher dust weight levels in U.S. households. Higher levels of dust weight were associated with greater odds of current asthma and wheeze. The strongest associations were seen for wheeze [adjusted odds ratio (OR) = 1.99; 95% confidence interval (CI), 1.21–3.28 for bedroom bed dust; OR = 2.81; 95% CI, 1.52–5.21 for upholstery dust). These associations persisted when adjusting for allergen and endotoxin exposures.

**Conclusions:**

Dust weight, an index of total dust exposure in the home, may contribute to respiratory outcomes independently of the exposure to specific components.

Since house dust was recognized as a common respiratory allergen in the early 20th century, research has focused on identifying the specific causes of its allergenicity. Early hypotheses made distinctions between settled house dust and “street dust,” proposing that decayed cotton “linters” and “kapok fibers” from household furnishings and carpets were the allergenic agents in house dust [described by Sanghvi et al. (1958)]. Later, experiments suggested that allergic reactions were caused by a biological interaction between kapok fibers and the mold extracted from them, but not by either agent alone [described by Jaggi and Viswanathan (1965)]. Refinement of laboratory extraction and purification techniques led to the identification of several active protein fractions of house dust (Versie et al. 1966). Finally, the discovery of *Dermatophagoides pteranyssinus*, the “house dust mite” (HDM), by Voorhorst et al. (1964) resulted in a pause in the search for the putative agent and accelerated research into the distribution and characteristics of this arthropod and its fragments. During this flurry of descriptive research, one author described the discovery of HDM as a “new and refreshing idea” (Unger 1967), and another described the search for the allergenic agent as “tantalizing and, until recently, frustrating” (Mitchell et al. 1969).

The discovery of HDM did not cause research into the allergenic properties of house dust to cease completely, however, because people continued to react to dust, even when it did not contain dust mites (Kern 1970). Furthermore, other allergenic agents, such as cockroach, pollen, and fungi, were identified in dust (Bernton and Brown 1970; Sinha et al. 1970). Over the past 35 years, many specific allergenic proteins were identified, and methods to quantify their concentrations were developed. The proposed increase in asthma, allergic sensitization, and allergic diseases since 1980 has renewed the “tantalizing” aspect of the search for specific household exposures associated with the etiology and exacerbation of these diseases, albeit with an emphasis on the biologically relevant concentrations of allergens, in addition to the allergens themselves. Although allergens have been emphasized in studies of house dust and allergic diseases, their concentrations, typically measured in micrograms per gram of dust, likely make up a very small fraction of dust. Dust is a heterogeneous mixture comprising a variety of inorganic and organic particles, metals, and fibers of different sizes. Occupational studies have shown that many nonallergenic particles in dust can exacerbate asthma. In addition, research has shown that activities that disturb dust reservoirs can increase exposures to airborne particles, such as particulate matter (PM) < 2.5 μm in aerodynamic diameter (PM_2.5_), PM < 5 μm (PM_5_), and PM < 10 μm (PM_10_), which have been linked to asthma.

The National Institute of Environmental Health Sciences and the U.S. Department of Housing and Urban Development conducted the National Survey of Lead and Allergens in Housing (NSLAH), from 1998 to 1999, to assess household exposures to allergens in homes representative of the noninstitutionalized U.S. population (Vojta et al. 2002). The survey obtained information on housing characteristics and occupants’ health via questionnaire. Vacuumed dust samples, which have generally been used in studies to assess household exposures, were collected to measure concentrations of a variety of allergens and endotoxin. The allergen assays included cockroach allergen Bla g 1, dust mite allergens Der f 1 and Der p 1, cat allergen Fel d 1, dog allergen Can f 1, mouse allergen Mus m 1, and allergens of the fungus *Alternaria alternata.* The purpose of the present study was to revisit the importance of dust per se as a respiratory allergen, taking into account the presence of specific allergenic agents that have been identified over the past 40 years. We used dust weight as an index of total dust exposure. A second goal was to describe the distribution and predictors of dust weight across different household sites in a nationally representative sample of homes in the United States.

## Methods

### Study design and data

The data for this study were obtained from the NSLAH, a cross-sectional survey designed to represent the national housing stock of approximately 96 million permanently occupied, noninstitutional housing units that permit resident children. Detailed descriptions of the study design, methodology, and response rates are available elsewhere (Jacobs et al. 2002; Vojta et al. 2002). Briefly, 831 housing units inhabited by 2,456 individuals were surveyed in 75 locations across the United States. In each household, a questionnaire was administered to an adult representative living in the home, vacuumed dust samples were collected, and observations about household characteristics were recorded, after the adult representative gave informed consent. The study protocol was approved by the National Institute of Environmental Health Sciences Institutional Review Board in 1998.

### Asthma and wheeze

We defined current asthma as self-reported physician-diagnosed asthma and either a report of asthma symptoms in the past year or current use of medication for asthma. Current wheeze was characterized as wheezing or whistling in the chest in the past year. We calculated prevalence estimates for asthma and wheezing among households with at least one dust sample and with complete information on asthma and wheezing among the occupants. Dust samples were available for 829 households, and information on asthma and wheezing was available for 2,439 (99.3%) and 2,319 (94.4%) individuals, respectively.

### Exposure assessment

We collected single surface dust samples from the following five locations in the home, using well-defined protocols (Vojta et al. 2002): kitchen floor; living room sofa or chair; living room floor; bedroom bed of the youngest child in the home; or from a randomly selected bed if no children were in the home; bedroom floor. Each sampling site was vacuumed for 5 min using the Eureka Mighty-Mite 7.0-ampere vacuum cleaner (Eureka Company, Bloomington, IL). A 19 mm × 90 mm cellulose extraction thimble (Whatman International, Ltd., Maidstone, UK) was placed into the distal end of the vacuum extension tube and sealed with a rubber O-ring gasket, and a clean crevice device tool was placed over the distal end of the tube.

Dust samples were sealed in resealable plastic bags and shipped overnight to a field office, where they were stored at –20°C until further use. Dust was sieved through a 425-μm pore-sized grating, and the recovered dust was weighed. Samples were analyzed for allergen content by methods detailed elsewhere (Vojta et al. 2002).

### Statistical methods

Dust weight was log-transformed to achieve a normal distribution for analyses (Kleinbaum et al. 1998). In addition to the five site-specific dust weights, we calculated two household indices: one to represent the average dust weight in the household (i.e., geometric mean dust weight across the sites available) and the other to represent the maximum dust weight in the household. To maximize the number of samples in the analyses, 0.01 was added to the dust weight for locations reporting zero dust weight.

We reported dust as total mass, in milligrams, for each household site and for household indices because we did not have information on the area of all vacuumed sites. However, we compared results of analyses using household indices based on all sites with results using indices based only on sites for which load per unit area could be calculated, and found that they were similar. Given these exploratory analyses, we determined that total dust mass would be a reasonable measure for exposure. Samples were present for all locations from 91% of the houses.

For descriptive purposes, we constructed models to identify predictors of household and site-specific dust weights. We identified predictors of household mean dust weight and site-specific dust weights through multivariable linear regression models using a backward elimination procedure. Any variables having a Wald effect test *F*-statistic with a *p*-value > 0.50 were removed from the initial full model in the first step. Additional variables were removed from the model in an iterative process that alternated refitting the model and removing the least predictive variable in the model until all of the remaining variables were statistically significant (*p* ≤0.05).

In the prediction models for household-level dust weights, we included variables that could be summarized for the entire house [Table S1, Supplemental Material (http://www.ehponline.org/docs/2006/9412/suppl.pdf)]. In prediction models for site-specific dust weights, we included variables that were specific to each room, (e.g., floor type, measured humidity and temperature, and observation of moisture in the room) (Table S2, Supplemental Material).

For analyses related to health outcomes, we calculated odds ratios (ORs) and 95% confidence intervals (CIs) using logistic regression. We used a change-in-estimate method to evaluate variables as confounders, using a cutoff criterion of 10% change in the ORs for asthma or wheeze (Greenland 1989). We adjusted logistic regression models for education, race, environmental tobacco smoke, sex, and age. Adjusting for other potential confounders, including the construction year of house, family income, and presence of pets, did not change the ORs appreciably. Because the results were not modified by age, we did not stratify our results by age groups (children vs. adults).

We entered mean and maximum house indices for allergen concentrations (Bla g 1, Der f 1 + Der p 1, Fel d 1, Can f 1, Mus m 1, and *Alternaria alternata*) into the logistic models to assess their effects on the relationship between dust weight and respiratory outcomes. We did not use site-specific allergen concentrations because sample sizes were compromised due to some missing data. Because allergen concentrations are dependent on dust levels, we used a relative measure of allergen concentration (allergen level per milligram dust) in the models.

We developed standard errors (SE), CIs, and *p-*values in accordance with the complex survey design using Taylor series linearization methods. We used general estimating equations to account for clustering of individuals within households. All percentages, means, percentiles, and ORs were weighted to represent the U.S. population of permanently occupied, noninstitutional housing units that permit resident children.

A detailed description of the statistical weighting for the NSLAH can be found elsewhere (Vojta et al. 2002). Statistical analyses were conducted using SUDAAN (release 9.01; Research Triangle Institute, Research Triangle Park, NC) and SAS (version 9.1; SAS Institute Inc., Cary, NC).

## Results

There were 2,456 individuals in the study population, living in 829 homes with dust samples. Most were female, white, and non-Hispanic (Table 1). In the study population, 6.9% reported current asthma, 11.2% reported ever-diagnosed asthma, and 15.9% experienced wheezing in the past year. Of the individuals with current asthma, 71% reported current use of medications for asthma. Individuals who reported doctor-diagnosed allergies (25.4%) were more likely to have current asthma (OR = 10.9; 95% CI, 7.3–16.2) and wheeze (OR = 4.2; 95% CI, 3.3–5.5) than those who had not been diagnosed with allergies.

### Distribution of dust weight

**Figure 1:**
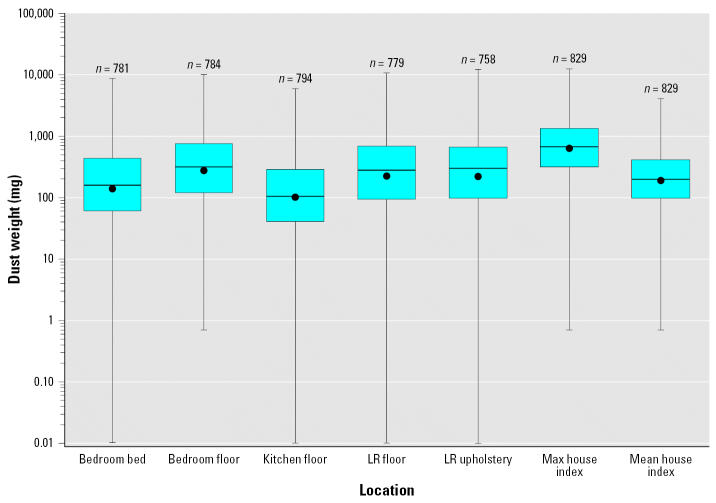
Distribution of dust weight (mg) by location in households, from the NSLAH, 1998–1999. Abbreviations: LR, living room; Max, maximum. Each box extends from the 25th to the 75th percentiles, and the error bars mark the maximum and minimum nonzero value. The dot represents the geometric mean for each location, and the horizontal line represents the median.

Of the five sampled sites in the home, the bedroom floor had the highest mean dust weight [geometric mean (GM) 279.1 mg, geometric standard error (GSE) 19.6], whereas the kitchen floor had the lowest (GM 111.1 mg, GSE 8.7). The living room upholstery had the widest range of dust weight (0–12215.2 mg), although the distributions for all sites were generally similar (Figure 1). The GMs for the house indices based on mean and maximum dust weights were 200.6 mg (GSE 12.6) and 644.7 mg (GSE 37.0), respectively. Dust weights were significantly correlated between sampling sites, with Spearman correlation coefficients ranging from 0.31 to 0.58 (Table 2).

### Predictors of dust weight

Several characteristics were associated with house dust weight (Table 3). [The complete list of variables evaluated in the linear regression model may be viewed in Table S1 in the Supplemental Material (http://www.ehponline.org/docs/2006/9412/suppl.pdf).] Lower income, older home construction, having pets or a smoker in the home, and less frequent cleaning of the living room floor were associated with higher household mean dust weights. Similar results were obtained using household maximum dust weight (Table 3). All of these variables, except having a smoker in the home, were also predictive of dust levels in most household sites, although the construction year of the home was the only variable that remained in every prediction model (Table 4). Smoking was statistically significantly associated with dust weight in site-specific analyses, although it did not remain in the final prediction model. Race was associated with dust weight in several sites: Black race predicted higher dust weights from living room and kitchen floors, whereas white race predicted dust weight in living room upholstery (Table 4). Higher education (≥high school) predicted dust weight in living room upholstery.

As expected, the presence of carpet predicted dust weight for floor sites, whereas cleaning variables, higher room humidity, air conditioning, and observed moisture remained in some but not all floor-specific prediction models. Predictors unique to dust levels in the bedroom bed included the number of stories in the house, the presence of mattress covers, and the absence of stuffed animals on the bed. Prediction variables unique to dust levels in the living room upholstery included room temperature, number of people in the home, education, and cleaning frequency for the upholstery. Dust weights for all factors considered in the site-specific models, and coefficients from linear regression models, may be viewed in Tables S2 and S3 of the Supplemental Material, respectively (http://www.ehponline.org/docs/2006/9412/suppl.pdf).

### Dust weight in relation to asthma and wheeze

Higher dust weights were associated with an approximately 2-fold increase in odds of having current asthma and wheeze, when the household index based on maximum dust weights was considered as exposure (Table 5). Most site-specific exposures, particularly the bedroom bed and the floors of the living room and kitchen, were associated with asthma and wheeze. Higher dust levels from living room upholstery were more clearly associated with wheezing than with asthma. Dust weights from the bedroom floor were not associated with either asthma or wheeze. Adjustment for income, construction year of home, and presence of pets did not change the ORs substantially, and we excluded these variables from the final adjusted model. Dust weight was associated with wheeze and asthma even when analyses were stratified by allergic status (defined as doctor-diagnosed allergies); however, there was no evidence of effect modification by allergic status (*p*-values for interaction > 0.10) [Table S4, Supplemental Material (http://www.ehponline.org/docs/2006/9412/suppl.pdf)].

In analyses stratified by urban/rural status, higher dust weights were associated with both asthma and wheeze irrespective of the location of the residence [Table S5, Supplemental Material (http://www.ehponline.org/docs/2006/9412/suppl.pdf)]. When maximum house dust was used as the exposure measure, however, the odds of wheeze were higher for those with an urban residence compared with a nonurban residence (*p*-value for interaction = 0.05). 

Because dust collected from one bedroom in each home was used to characterize exposure for all members of the household, we limited analyses to the 126 households with one occupant. In these models, the positive relationship between dust weight and respiratory outcomes was of a greater magnitude, although precision was lost due to smaller sample size. For example, the adjusted ORs for individuals in the highest quartile of bedroom bed dust weight, compared with those in the lowest quartile, were 7.40 (95% CI, 1.4–40.4) for one-occupant households and 1.76 (95% CI, 1.02–3.04) for all households. We repeated the logistic regression models using dust weight as a continuous variable and found positive associations with asthma, with statistically significant associations for the bedroom bed (*p* = 0.02), the kitchen floor (*p* = 0.03), and the mean house index (*p* = 0.02).

### Effects of allergens and endotoxin

Introduction of allergens and endotoxin into the models did not change the results appreciably [Table S6, Supplemental Material (http://www.ehponline.org/docs/2006/9412/suppl.pdf)]; however, the positive association between dust weight and asthma prevalence was strengthened when Bla g 1 and Mus m 1 allergen were added to the models. The allergens considered were Bla g 1, Der f 1, Der p 1, Fel d 1, Can f 1, Mus m 1, and *Alternaria alternata.* Spearman rank correlations between dust weight and allergen concentrations were negligible (data not shown).

## Discussion

We examined the relationship between household dust and asthma symptoms among participants in the NSLAH and found that respiratory symptoms were associated with higher levels of dust weight. Asthma and wheeze were reported twice as often by individuals in households with the greatest amount of dust than by those with the least amount of dust, when exposure was based on household indices of maximum and mean dust weights. When dust weights from specific household sites were considered, the bedroom bed was associated with asthma and wheeze, whereas the living room upholstery was associated with wheeze. These relationships held after adjustment for potential confounders, including concentrations of common indoor allergens and endotoxin that have generally been associated with asthma symptoms.

In the study population, 6.9% reported current asthma, 11.2% reported ever-diagnosed asthma, and 15.9% experienced wheezing in the past year. These prevalence estimates were comparable to other national surveys; for example, the National Health Interview Survey and the Behavioral Risk Factor Surveillance System reported 7.0% and 7.7% current asthma prevalence and 10.4% and 11.9% lifetime asthma prevalence, respectively (Centers for Disease Control and Prevention 2003, 2005).

We identified predictors of dust weight at the household level and for individual household sites. Lower income, home construction year before 1946, having pets, having a smoker in the home, and less frequent cleaning of the living room carpet were associated with higher household levels of dust. Home construction year predicted dust levels for all five household sites. Lower income and the presence of pets predicted dust for most sites, and cleaning variables specific to each site were common predictors of dust weight for those sites. For example, not cleaning floors in the kitchen and living room within the previous week was predictive of higher dust weights vacuumed from those sites. Site-specific models identified other predictors, including the presence of carpet, room humidity, observed moisture, region of the country, and lack of air conditioning. Because we included many variables into the prediction models, it is possible that the statistical significance of some predictors occurred by chance. However, to minimize this possibility, we included only variables that might plausibly affect levels of dust, some of which have been identified in previous studies.

In this study we describe dust weight as an important factor in respiratory symptoms, apart from the independent effects of specific allergens. Most recent studies have regarded specific allergens, such as Der p 1, Der f 1, Fel d 1, and Bla g 1, as the important agents in the etiology and exacerbation of asthma (Custovic et al. 1998; Lau et al. 2000; Sporik et al. 1990). Although many studies have focused on identifying specific allergens, other researchers have raised the question of whether asthma is strictly an allergen-mediated disease (Arshad et al. 2001; Pearce et al. 1999, 2000). Interest in this area has expanded research since the 1990s, with improved exposure assessment and investigation into other agents that may be associated with the onset or exacerbation of asthma. For example, associations have been found between asthma symptoms and exposure to phthalates (Bornehag et al. 2004; Hoppin et al. 2004), pesticides (Salam et al. 2004), cigarette smoke, endotoxin (Thorne et al. 2005), and outdoor pollutants (King et al. 2004; Wallace et al. 2003).

The relationship between dust and asthma has been highlighted in the occupational literature, where asthma is also a significant concern. In these studies, asthma has been associated with exposures to organic (e.g., flour, wood, and grains) and inorganic (e.g., silicates) dusts in a variety of occupations (Baur et al. 1998; Brant et al. 2005; Kirkhorn and Garry 2000; Zock et al. 2004). In response to concerns about respiratory symptoms caused by exposure to a variety of dusts, the Occupational Safety and Health Administration (OSHA) set a permissible limit for nuisance dust in the workplace (OSHA 1993). The standard (29 CFR 1910.1000) limits employee exposure to 15 mg/m^3^ air averaged over an 8-hr work shift, measured as total dust. If only the respirable fraction is measured, the exposure is limited to 5 mg/m^3^ air.

A separate body of literature addresses the biologic activity of house dust as a whole rather than looking at specific components (Roberts and Dickey 1995). One study conducted in Copenhagen schools found that dust with high potency to stimulate interleukin secretion from lung epithelial cells was associated with generalized symptoms of fatigue as well as symptoms of the eyes, nose, throat, and skin (Allermann et al. 2003). Similarly, dust samples from residences in Sweden stimulated strong interleukin responses, suggesting that house dust contains one or more potent agents that may cause or exacerbate respiratory disease (Saraf et al. 1999). The studies focusing on the overall content of dust tend to recognize dust as a heterogeneous mixture comprising a variety of inorganic and organic particles and fibers of different sizes, rather than a repository for one specific allergen or exposure (Butte and Heinzow 2002).

Our results indicated that asthma symptoms were more consistently associated with dust weight collected from the bed than from other household sites. It is reasonable to suspect that the bed is a significant source of exposure considering the amount of time spent there and the proximity of the breathing zone to the mattress and pillow, which were the sampled locations in this study. It is also possible that finer dust particles settle on the bed and are easily resuspended, thereby promoting short-term high-intensity exposures (when a person sits on or enters the bed) as well as pervasive exposures of small particles that remain airborne but enter the lower airways when respired. This is consistent with other research that has shown that activities that disturb dust reservoirs on furniture or on textiles increase exposures of PM_2.5_, PM_5_, and PM_10_ substantially (Ferro et al. 2004). This may also explain why we found an association between dust levels from the living room upholstery and wheeze. Interestingly, levels in the upholstery were not associated with asthma. A possible explanation might be that doctor-diagnosed asthmatics are likely to be more familiar with asthma triggers than undiagnosed individuals, and households with diagnosed asthmatics may be less likely to have dust-gathering upholstery.

Our findings agree with other research showing relationships between bed dust exposures and respiratory symptoms, although many of the previous studies have focused on dust mite allergens (Garrett et al. 1998; Marks et al. 1995a, 1995b; van den Bemt et al. 2004). We considered that symptoms may have been related to allergens or endotoxin, which was our rationale for including allergen and endotoxin concentrations into logistic regression models, but their inclusion did not change the ORs appreciably.

Asthma and wheeze were associated with elevated levels of dust from the kitchen and living room floors. Perhaps these sites have characteristics that set them apart from the bedroom floor, which was not associated with asthma or wheeze in this study. For example, compared with bedrooms, family rooms and kitchens may have more people in them at one time, disturbing settled dust. It is not uncommon for children to play on the floors of these rooms while family members are busy with chores or activities, which may also confer exposure. Differences between rooms, such as temperature, humidity, moisture, number of windows, floor type, or ventilation may also influence the degree of exposure.

The lack of biological outcome data, such as skin prick tests, specific immunoglobulin E, or lung function tests, necessitates dependence on questionnaire data for asthma diagnosis and reporting of respiratory or allergy symptoms. Self-reported health outcomes are subject to bias, such as inability to recall information or lack of knowledge. However, the use of questionnaire data has been found to be quite reliable in identification of wheeze and asthma (Eggleston et al. 2005; Jenkins et al. 1996). Moreover, prevalence of asthma and wheeze in our survey were comparable with other large national surveys (Centers for Disease Control and Prevention 2003, 2005).

We acknowledge that dust samples collected from the bedrooms were not matched at the level of the individual—that a reported asthmatic or wheezing household member may not have been the occupant of the bedroom that was sampled. Neither can we determine whether the reported wheezing household members spent time on the living room furniture. It is likely, however, that most family members spend time on the “most used” furniture in the living room. We believe the strong associations found between wheeze and dust from the living room furniture lends credibility to the associations found between respiratory symptoms and dust from the bed, in that it is likely that dust weight from the sampled bedroom represents dust weight in other bedrooms of the home. Furthermore, our results were strengthened when we limited analyses to one-occupant households where bed exposure can be attributed unequivocally to the individual.

A major strength of this study is its complex, multistage sampling strategy, designed to represent the broader U.S. population. This is in contrast to other studies that have focused on specific areas, such as innercity or rural homes. This study also provides information about dust levels across different household sites, rather than a particular site such as the bedroom bed, giving a more complete picture of household exposure.

In summary, we found an association between dust levels and asthma symptoms even after controlling for common indoor allergens and endotoxin. This probably means that there are other unmeasured irritants or sensitizers in dust, such as pesticides or unidentified allergens, which adversely affect respiratory health. This would be reasonable to expect, because dust is a “sink” for semivolatile organic compounds, particulate organic matter, and metals (Butte and Heinzow 2002). Another interpretation is that dust itself may have irritant properties that induce inflammation in the lungs, consistent with air pollution literature that suggests that exposure to small particles may have adverse health effects (Seaton et al. 1995). In either case, it is clear that dust should be considered a significant household exposure in studies of risk assessment for respiratory disease, because it not only gives an indirect measure of the particular agent (e.g., allergen load), but also gives an indirect measure of anything else that might be present in the household environment.

## Figures and Tables

**Table 1:** Characteristics of the study population from the NSLAH, 1998–1999.

Characteristic	Frequency (*n* = 2,456)	Weighted*a* frequency (*n* = 260,515) (× 1,000)	Weighted percent
Male	1,189	125,123	48.0
Female	1,256	134,636	51.7
Hispanic	338	26,560	10.3
Non-Hispanic	2,088	230,505	89.7
White	1,788	204,784	78.6
Black	355	29,777	11.4
Other	262	21,706	8.3
Age (years)			
0–10	481	41,479	16.3
11–20	396	40,156	15.7
21–30	329	33,201	13.0
31–40	425	46,110	18.1
41–50	344	39,269	15.4
51–60	215	25,275	9.9
61–70	110	14,835	5.8
≥71	105	14,688	5.8
Smoking			
Yes	1,012	107,292	41.2
No	1,430	151,413	58.1
Household education^b^			
< High school	214	19,878	7.9
High school	519	53,230	21.3
> High school	1,623	176,996	70.8
Asthma	174	—	6.9
Wheezing	353	—	15.9

a Weighted for the multistage sampling design of the NSLAH.

b Highest education attained in the household.

**Table 2:** Spearman correlation coefficients between dust weight levels across sampled sites, from the NSLAH, 1998–1999 (all *p*-values < 0.0001).

Location	BR bed	BR floor	LR floor	LR sofa/chair	Kitchen floor	House mean
BR floor	0.37	—	—	—	—	—
LR floor	0.38	0.58	—	—	—	—
LR sofa	0.38	0.35	0.38	—	—	—
Kitchen floor	0.30	0.40	0.44	0.31	—	—
House mean	0.66	0.75	0.77	0.67	0.67	—
House maximum	0.55	0.70	0.71	0.59	0.53	0.84

Abbreviations: BR, bedroom; LR, living room.

**Table 3:** Geometric means of dust weight (mg) for predictors^a^ of dust weight at the household level, from the NSLAH, 1998–1999.

	No. of homes	House index based on mean GM [GSE (mg)]	*p*-Value^b^	House index based on maximum GM [GSE (mg)]	*p*-Value
Household income (US$)					
0–19,999	188	276.0 (28.3)	< 0.001	919.0 (90.4)	< 0.001
20,000–39,999	227	228.3 (19.2)		753.8 (52.7)	
40,000–59,999	152	177.1 (23.6)		584.9 (64.1)	
≥60,000	203	116.1 (12.6)		393.1 (41.9)	
House construction year					
1978–1998	220	157.6 (14.1)	0.006	515.4 (45.7)	0.0003
1960–1977	267	159.7 (13.7)		549.8 (45.1)	
1946–1959	141	225.2 (28.7)		720.7 (84.2)	
1940–1945	44	319.0 (51.9)		1003.1 (130.2)	
1939 or earlier	157	287.3 (33.5)		961.8 (100.1)	
Pets in the home					
Yes	400	208.4 (18.1)	0.034	679.8 (52.5)	0.047
No	421	184.5 (14.1)		621.1 (44.9)	
Smoker in the home					
Yes	332	233.8 (17.9)	0.002	790.5 (59.6)	0.040
No	493	173.4 (12.6)		567.7 (37.3)	
Last time living room floor/carpet cleaned					
< 1 week	484	176.8 (13.5)	0.050	612.5 (43.3)	0.264
≥1 week	313	207.6 (13.8)		652.5 (42.8)	

a From multivariable linear regression models.

b *p*-Value for *F*-test from linear regression model predicting dust weight.

**Table 4:** Predictors of dust weight^a^ for each household site, resulting from multivariable linear regression models (NSLAH, 1998–1999). Regression coefficients may be viewed in the Supplemental Material (Table S3).

	Bedroom bed	Bedroom floor	Kitchen floor	LR floor	LR sofa
Variables entered into every site-specific model					
Older construction year	↑	↑	↑	↑	↑
Region of country					
Northeast	↓	↓		↓	
Midwest	↓	↓		↑	
South (reference = West)	↓	↑		↑	
Urbanization			↓		
Increasing stories in house	↑				
Air conditioning in home		↓		↓	
Observed moisture in room	↑	↑	↑		
Lower humidity		↑	↑		
Higher temperature in room					↓
More people in home					↑
Pets in home		↑	↑	↑	↑
Lower household income	↑	↑		↑	↑
White race		↓	↓		↑
≥High school education					↑
Variables entered into selected site-specific models					
Floor cleaned < 1 week ago^b^		↓		↓	
Presence of carpet^b^		↑	↑	↑	
Upholstery cleaned > month ago^c^					↑
Mattress covers^d^	↓				
Stuffed animals on bed^d^	↓				

LR, living room.

a Direction of arrow indicates increased or decreased dust weight associated with variable.

b Entered only into floor-related models.

c Entered only into upholstery-related model.

d Entered only into bed-related model.

**Table 5:** Unadjusted and adjusted ORs^a^ for current asthma and wheeze, for quartiles of dust weight, by house location and index, from the NSLAH, 1998–1999.

	Asthma (*n* = 174)		Wheeze (*n* = 353)	
Locations (quartiles)^b^	Crude OR (95% CI)	Adjusted OR (95% CI)	Crude OR (95% CI)	Adjusted OR (95% CI)
Bedroom bed				
2nd	1.10 (0.61–1.99)	1.23 (0.63–2.40)	1.41 (0.87–2.28)	1.42 (0.82–2.47)
3rd	1.35 (0.74–2.45)	1.42 (0.72–2.78)	1.52 (0.93–2.48)	1.29 (0.79–2.11)
4th	1.76 (1.02–3.04)	1.89 (1.09–3.27)	2.21 (1.37–3.56)	1.99 (1.21–3.28)
Bedroom floor				
2nd	0.99 (0.53–1.85)	0.83 (0.41–1.68)	0.96 (0.46–2.00)	0.87 (0.40–1.92)
3rd	0.95 (0.49–1.85)	0.88 (0.43–1.80)	0.86 (0.53–1.37)	0.79 (0.48–1.29)
4th	1.00 (0.60–1.64)	0.92 (0.53–1.57)	1.47 (0.89–2.43)	1.46 (0.86–2.48)
Kitchen floor				
2nd	0.87 (0.44–1.71)	0.97 (0.49–1.92)	1.40 (0.88–2.23)	1.51 (0.91–2.49)
3rd	1.31 (0.67–2.57)	1.28 (0.64–2.54)	1.68 (0.99–2.86)	1.68 (0.99–2.86)
4th	1.65 (0.85–3.21)	1.92 (1.03–3.60)	1.24 (0.77–2.02)	1.30 (0.78–2.18)
LR floor				
2nd	0.60 (0.32–1.16)	0.65 (0.30–1.42)	1.07 (0.67–1.70)	1.19 (0.75–1.89)
3rd	0.81 (0.46–1.45)	0.83 (0.44–1.59)	1.49 (1.07–2.06)	1.55 (1.07–2.25)
4th	1.39 (0.94–2.05)	1.52 (1.01–2.29)	1.44 (0.91–2.29)	1.51 (0.94–2.44)
LR upholstery				
2nd	1.29 (0.73–2.26)	1.30 (0.69–2.45)	1.30 (0.81–2.09)	1.27 (0.73–2.19)
3rd	0.64 (0.32–1.27)	0.63 (0.30–1.33)	1.22 (0.72–2.07)	1.26 (0.72–2.21)
4th	0.93 (0.56–1.56)	0.98 (0.59–1.62)	2.42 (1.32–4.46)	2.81 (1.52–5.21)
Index (mean)				
2nd	1.55 (0.82–2.93)	1.27 (0.52–3.15)	1.10 (0.71–1.70)	0.99 (0.58–1.69)
3rd	1.44 (0.74–2.81)	1.58 (0.77–3.28)	1.92 (1.19–3.07)	1.98 (1.12–3.50)
4th	1.88 (1.03–3.44)	1.79 (0.91–3.51)	1.74 (1.09–2.79)	1.59 (1.06–2.38)
Index (maximum)				
2nd	1.47 (0.72–3.02)	1.57 (0.81–3.03)	1.33 (0.75–2.35)	1.41 (0.77–2.60)
3rd	1.70 (0.81–3.58)	1.93 (0.89–4.18)	2.45 (1.41–4.25)	1.96 (1.22–3.17)
4th	1.96 (1.02–3.78)	2.21 (1.08–4.55)	2.15 (1.43–3.24)	1.81 (1.18–2.75)

LR, living room.

a Adjusted for sex, age (categorized in decades), race, education, and environmental tobacco smoke exposure.

b Reference = 1st quartile.

## References

[b1-ehp0115-000215] Allermann L, Meyer HW, Poulsen OM, Nielsen JB, Gyntelberg F (2003). Inflammatory potential of dust from schools and building related symptoms. Occup Environ Med.

[b2-ehp0115-000215] Arshad SH, Tariq SM, Matthews S, Hakim E (2001). Sensitization to common allergens and its association with allergic disorders at age 4 years: a whole population birth cohort study. Pediatrics.

[b3-ehp0115-000215] Baur X, Chen Z, Liebers V (1998). Exposure-response relationships of occupational inhalative allergens. Clin Exp Allergy.

[b4-ehp0115-000215] Bernton HS, Brown H (1970). Insect allergy: the allergenicity of the excrement of the cockroach. Ann Allergy.

[b5-ehp0115-000215] Bornehag C-G, Sundell J, Weschler CJ, Sigsgaard T, Lundgren B, Hasselgren M (2004). The association between asthma and allergic symptoms in children and phthalates in house dust: a nested case–control study. Environ Health Perspect.

[b6-ehp0115-000215] Brant A, Berriman J, Sharp C, Welch J, Zekveld C, Nieuwenhuijsen M (2005). The changing distribution of occupational asthma: a survey of supermarket bakery workers. Eur Respir J.

[b7-ehp0115-000215] Butte W, Heinzow B (2002). Pollutants in house dust as indicators of indoor contamination. Rev Environ Contam Toxicol.

[b8-ehp0115-000215] Centers for Disease Control and Prevention 2003. Behavioral Risk Factor Surveillance System. Hyattsville. MD:National Center for Health Statistics.

[b9-ehp0115-000215] Centers for Disease Control and Prevention. 2005. National Health Interview Survey 2005. Hyattsville, MD:National Center for Health Statistics.

[b10-ehp0115-000215] Custovic A, Smith A, Woodcock A (1998). Indoor allergens are a primary cause of asthma. Eur Respir J.

[b11-ehp0115-000215] Eggleston PA, Diette G, Lipsett M, Lewis T, Tager I, McConnell R (2005). Lessons learned for the study of childhood asthma from the Centers for Children’s Environmental Health and Disease Prevention Research. Environ Health Perspect.

[b12-ehp0115-000215] Ferro AR, Kopperud RJ, Hildemann LM (2004). Elevated personal exposure to particulate matter from human activities in a residence. J Expo Anal Environ Epidemiol.

[b13-ehp0115-000215] Garrett M, Hooper B, Hooper M (1998). Indoor environmental factors associated with house-dust-mite allergen (Der p 1) levels in south-eastern Australian houses. Allergy.

[b14-ehp0115-000215] Greenland S (1989). Modeling and variable selection in epidemiologic analysis. Am J Public Health.

[b15-ehp0115-000215] Hoppin JA, Ulmer R, London SJ (2004). Phthalate exposure and pulmonary function. Environ Health Perspect.

[b16-ehp0115-000215] Jacobs DE, Clickner RP, Zhou JY, Viet SM, Marker DA, Rogers JW (2002). The prevalence of lead-based paint hazards in U.S. housing. Environ Health Perspect.

[b17-ehp0115-000215] Jaggi OP, Viswanathan R (1965). House dust allergy. J Indian Med Assoc.

[b18-ehp0115-000215] Jenkins MA, Clarke JR, Carlin JB, Roberston CF, Hopper JL, Dalton MF (1996). Validation of questionnaire and bronchial hyperresponsiveness against respiratory physician assessment in the diagnosis of asthma. Int J Epidemiol.

[b19-ehp0115-000215] Kern RA (1970). Mites as allergens in house dust. JAMA.

[b20-ehp0115-000215] King ME, Mannino DM, Holgiun F (2004). Risk factors for asthma incidence. A review of recent prospective evidence. Panminerva Med.

[b21-ehp0115-000215] Kirkhorn SR, Garry VF (2000). Agricultural lung diseases. Environ Health Perspect.

[b22-ehp0115-000215] Kleinbaum DG Kupper LL Muller KE Nizam A 1998. Applied Regression Analysis and Other Multivariable Methods. Pacific Grove, CA:Duxbury Press.

[b23-ehp0115-000215] Lau S, Illi S, Sommerfeld C, Niggemann B, Bergmann R, von Mutius E, Wahn U (2000). Early exposure to house-dust mite and cat allergens and development of childhood asthma: a cohort study. Lancet.

[b24-ehp0115-000215] Marks BGB, Tovey ER, Green W, Shearer M, Salome CM, Woolcock AJ (1995a). The effect of changes in house dust mite allergen exposure on the severity of asthma. Clin Exp Allergy.

[b25-ehp0115-000215] Marks BGB, Tovey ER, Toelle BG, Peat JK, Woolcock AJ (1995b). Mite allergen (Der p 1) concentration in houses and its relation to the presence and severity of asthma in a population of Sydney schoolchildren. J Allergy Clin Immunol.

[b26-ehp0115-000215] Mitchell WF, Wharton GW, Larson DG, Modic R (1969). House dust, mites, and insects. Ann Allergy.

[b27-ehp0115-000215] OSHA (Occupational Safety and Health Administration) 1993. Occupational Safety and Health Standards 29 C.F.R. §1910.1000, Table Z-3.

[b28-ehp0115-000215] Pearce N, Douwes J, Beasley R (2000). Is allergen exposure the major primary cause of asthma?. Thorax.

[b29-ehp0115-000215] Pearce N, Pekkanen J, Beasley R (1999). How much asthma is really attributable to atopy?. Thorax.

[b30-ehp0115-000215] Roberts JW, Dickey P (1995). Exposure of children to pollutants in house dust and indoor air. Rev Environ Contam Toxicol.

[b31-ehp0115-000215] Salam MT, Li YF, Langholz B, Gilliland FD, Children’s Health Study (2004). Early-life environmental risk factors for asthma: findings from the Children’s Health Study. Environ Health Perspect.

[b32-ehp0115-000215] Sanghvi LM, Gupta KD, Sethi JP, Solomon SK, Kasliwal RM (1958). Significance of house dust as respiratory allergen. J Indian Med Assoc.

[b33-ehp0115-000215] Saraf A, Larsson L, Larrson B-M, Larsson K, Palmberg L (1999). House dust induces IL-6 and IL-8 response in A549 epithelial cells. Indoor Air.

[b34-ehp0115-000215] Seaton A, MacNee W, Donaldson K, Godden D (1995). Particulate air pollution and acute health effects. Lancet.

[b35-ehp0115-000215] Sinha RN, van Bronswijk JE, Wallace HA (1970). House dust allergy, mites and their fungal associations. Can Med Assoc J.

[b36-ehp0115-000215] Sporik R, Holgate T, Platts-Mills TAE, Cogswell JJ (1990). Exposure to house-dust mite allergen (Der p I) and the development of asthma in childhood. A prospective study. N Engl J Med.

[b37-ehp0115-000215] Thorne PS, Kulhankova K, Yin M, Cohn R, Arbes SJ, Zeldin DC (2005). Endotoxin exposure is a risk factor for asthma: The National Survey of Endotoxin in U.S. Housing. Am J Respir Crit Care Med.

[b38-ehp0115-000215] Unger L (1967). The house-dust mite. Ann Allergy.

[b39-ehp0115-000215] van den Bemt L, van Knapen L, de Vries MP, Jansen M, Cloosterman S, van Schayck CP (2004). Clinical effectiveness of a mite allergen-impermeable bed-covering system in asthmatic mite-sensitive patients. J Allergy Clin Immunol.

[b40-ehp0115-000215] Versie R, Monard Y, Geubelle F (1966). Comparative study of various extraction and purification procedures used for the preparation of house dust allergen. Int Arch Allergy Appl Immunol.

[b41-ehp0115-000215] Vojta PJ, Friedman W, Marker DA, Clickner R, Rogers JW, Viet SM (2002). First National Survey of Lead and Allergens in Housing: survey design and methods for the allergen and endotoxin component. Environ Health Perspect.

[b42-ehp0115-000215] Voorhorst R, Spieksma-Boezeman MI, Spieksma FT (1964). Is a mite (Dermatophagoides sp.) the producer of the house-dust allergen?. Allerg Asthma (Leipz).

[b43-ehp0115-000215] Wallace LA, Mitchell H, O’Connor GT, Neas L, Lippmann M, Kattan M (2003). Particle concentrations in inner-city homes of children with asthma: the effect of smoking, cooking, and outdoor pollution. Environ Health Perspect.

[b44-ehp0115-000215] Zock JP, Cavalle N, Kromhaut H, Kennedy SM, Sunyer J, Jaen A (2004). Evaluation of specific occupational asthma risks in a community-based study with special reference to single and multiple exposures. J Expo Anal Environ Epidemiol.

