# Supplementary material for: Dust Weight and Asthma Prevalence in the National Survey of Lead and Allergens in Housing (NSLAH)
Source: Environ Health Perspect. 2006 Nov 7;115(2):215–20. doi: 10.1289/ehp.9412 (PMC1817708; doi:10.1289/ehp.9412)
Supplement: Supplemental Figures and Tables [file ehp0115-000215s1.pdf]

Supplemental Material

Dust Weight and Asthma Prevalence in the National Survey of Lead and Allergens in Housing (NSLAH)

Leslie Elliott[1], Samuel J. Arbes[1], Eric S. Harvey[2], Robert C. Lee[2], Päivi M. Salo[1], Richard D. Cohn[2], Stephanie J. London[1], and Darryl C. Zeldin[1]

[1]Laboratory of Respiratory Biology, Division of Intramural Research, NIEHS, NIH, Research Triangle Park, NC

[2]Constella Group, LLC, Durham, NC

Corresponding Author:

Darryl C. Zeldin, M.D.
National Institute of Environmental Health Sciences
111 T.W. Alexander Drive, Building 101, D236
Research Triangle Park, NC 27709-2233
Phone: 919-541-1169
Fax: 919-541-4133
email: zeldin@niehs.nih.gov

Table S1. Dust Weight (mg) Summarized by Location and Levels of Factors

Factor		Total	Bedroom Bed			Bedroom Floor			Living Room Floor			Living Room Upholstery			Kitchen Floor			Geometric mean			Maximum		
		Sample Size **	Geometric Mean	Geometric mean SE	p-values	Geometric Mean	Geometric mean SE	p-values	Geometric Mean	Geometric mean SE	p-values	Geometric Mean	Geometric mean SE	p-values	Geometric Mean	Geometric mean SE	p-values	Geometric Mean	Geometric mean SE	p-values	Geometric Mean	Geometric mean SE	p-values
Household Income by 20K		770																					
	$0-19,999	188	192.77	20.49		430.73	57.72		383.22	60.17		300.44	46.48		149.52	18.77		276.02	28.28		919.00	90.44	
	$20,000-39,999	227	187.98	19.45	<.0001	348.93	38.09	<.0001	317.04	29.69	<.0001	292.62	34.34	<.0001	99.98	15.13	0.0554	228.31	19.18	<.0001	753.75	52.74	<.0001
	$40,000-59,999	152	126.84	34.52		257.34	26.92		211.13	27.83		247.20	28.17		129.48	22.68		177.11	23.63		584.90	64.06	
	$60,000+	203	92.38	11.98		151.60	25.12		123.17	19.81		140.42	16.72		85.21	12.78		116.12	12.58		393.09	41.85	
Season		829																					
	Winter	195	155.51	35.49		300.64	55.49		245.73	47.16		258.97	43.94		124.57	25.34		207.36	34.28		744.53	113.59	
	Summer	286	128.94	32.01	0.3889	222.94	28.61	0.1221	198.95	32.55	0.1721	222.74	34.28	0.7588	93.01	12.74	0.3598	161.86	23.41	0.2309	538.58	66.06	0.2693
	Fall	348	182.46	21.86		335.65	38.62		301.12	34.08		261.76	34.30		122.35	17.61		223.79	23.66		697.61	68.98	
Census Region		829																					
	Northeast	155	154.74	22.86		219.86	38.38		148.76	27.39		183.59	24.58		107.14	20.03		159.24	23.63		467.08	58.42	
	Midwest	196	165.97	27.05	0.9605	228.11	26.77	0.0510	280.76	30.88	0.0251	326.08	57.96	0.0683	126.48	19.84	0.8096	207.59	23.84	0.4379	657.78	73.38	0.0496
	South	277	144.34	30.63		327.91	42.54		282.85	33.77		228.85	32.79		108.25	14.65		199.89	25.18		748.73	83.50	
	West	201	153.97	11.64		334.33	44.23		261.21	55.32		267.62	40.70		103.61	15.56		210	20.41		660.73	60.74	
Urbanization		829																					
	MSA >= 1 million	274	154.48	11.27		233.52	24.23		215.29	22.15		194.43	20.13		92.38	10.18		169.11	12.36		576.06	47.30	
	MSA < 1 million	417	155.01	16.03	0.9894	272.78	24.99	0.1260	229.19	21.71	0.2420	237.36	26.12	0.0458	97.79	10.65	0.0093	186.50	14.01	0.2982	614.73	36.07	0.2308
	Non-MSA PSU	138	147.83	45.50		356.60	63.95		320.76	67.29		341.47	68.58		175.48	30.91		248.16	48.48		806.28	141.20	
Household Construction Year		829																					
	1978-1998	220	116.99	18.87		230.09	27.79		213.87	21.64		198.67	24.12		92.66	11.15		157.62	14.10		515.42	45.68	
	1960-1977	267	124.57	12.97		257.69	29.68		211.40	23.63		214.43	23.56		82.91	8.50		159.69	13.71		549.80	45.09	
	1946-1959	141	182.20	30.73	0.0092	270.85	48.85	0.0070	274.74	51.02	0.1355	309.51	41.48	0.0268	121.23	19.75	<.0001	225.18	28.73	0.0005	720.73	84.18	<.0001
	1940-1945	44	243.82	57.42		418.49	70.00		372.71	100.41		348.48	99.31		235.90	49.32		318.96	51.91		1003.11	130.20	
	1939 or earlier	157	257.80	37.92		405.37	62.40		300.26	50.95		314.56	50.15		172.04	21.96		287.34	33.5		961.79	100.09	
How many stories, including basement?		825																					
	1 story	330	125.40	25.12		308.88	40.89		280.14	37.76		257.21	32.28		113.34	15.39		194.83	25.78		674.37	84.10	
	2-3 stories	433	170.21	14.58	0.1013	263.04	25.87	0.5494	227.39	22.47	0.3895	238.54	23.76	0.8642	108.46	12.23	0.8724	194.93	14.00	0.9247	637.55	41.26	0.6742
	4 or more stories	62	208.83	25.79		254.41	40.62		205.85	45.65		233.38	49.59		121.01	21.66		191.06	23.97		557.13	90.70	

Factor	Total	Bedroom Bed			Bedroom Floor			Living Room Floor			Living Room Upholstery			Kitchen Floor			Geometric mean			Maximum		
	Sample Size **	Geometric Mean	Geometric mean SE	p-values	Geometric Mean	Geometric mean SE	p-values	Geometric Mean	Geometric mean SE	p-values	Geometric Mean	Geometric mean SE	p-values	Geometric Mean	Geometric mean SE	p-values	Geometric Mean	Geometric mean SE	p-values	Geometric Mean	Geometric mean SE	p-values
Main heating source in home?	826																					
Electric	230	148.07	18.02		318.03	29.11		254.71	28.68		222.25	31.21		99.49	9.25		195.12	14.83		666.75	51.46	
Gas	433	149.38	19.35	0.6756	261.62	30.33	0.3496	242.37	29.28	0.9052	255.13	28.91	0.7294	110.12	13.49	0.4736	189.87	20.14	0.8043	631.73	60.91	0.9031
Other/none	163	174.72	27.51		275.48	43.92		234.10	37.84		256.49	34.46		128.20	23.98		205.4	28.07		645.17	79.16	
Does home have air conditioning?	828																					
Air conditioning	653	144.09	15.38	0.0619	261.05	19.51	0.0523	229.86	18.21	0.0544	238.58	21.03	0.3677	105.52	8.76	0.1475	182.72	13.14	0.0429	613.54	40.72	0.0841
No air conditioning	175	201.99	31.09		379.62	65.63		317.27	50.31		275.52	39.48		143.14	27.09		256	34.29		801.44	105.23	
Air filtration device in home?	810																					
Yes	101	165.84	25.11	0.5303	244.56	34.81	0.3633	173.43	29.41	0.0299	222.96	34.45	0.5327	127.72	23.67	0.3631	186.46	21.30	0.5632	595.57	79.75	0.5767
No	709	150.57	15.06		283.85	22.09		254.29	19.97		246.37	20.49		107.66	8.54		194.04	13.4		645.83	39.71	
Dehumidifies used in home?	813																					
Yes	130	191.03	31.54	0.3003	203.75	24.22	0.0172	175.32	23.15	0.0083	310.83	62.52	0.1504	114.10	16.03	0.8626	188.37	18.65	0.6180	593.89	61.41	0.3959
No	683	152.70	15.75		299.00	25.49		262.54	21.67		234.58	17.53		110.92	9.74		199.18	14.46		660.79	43.29	
Any pets currently in house?	821																					
Yes	400	142.10	18.55	0.2292	286.40	22.01	0.7295	289.21	30.71	0.0103	266.58	27.17	0.1586	124.76	12.74	0.0572	208.38	18.11	0.3629	679.82	52.54	0.3453
No	421	166.85	17.98		276.61	26.39		214.42	18.33		228.81	20.65		99.97	9.65		184.48	14.08		621.05	44.93	
How many people live in house?	829																					
1 person	125	200.01	29.81		326.65	56.18		262.00	42.88		284.28	38.76		134.19	23.46		232.52	27.84		666.27	82.64	
2 people	253	176.69	27.50		269.61	28.02		242.47	21.70		246.25	33.00		100.18	13.49		190.54	16.64		684.81	52.59	
3 people	171	106.29	14.51	0.0040	266.12	34.17	0.8605	216.48	24.87	0.7733	187.48	17.12	0.0236	106.60	12.41	0.3953	172.52	13.19	0.2079	545.74	40.36	0.0885
4 people	165	138.48	22.40		273.52	34.15		258.63	36.53		275.43	34.25		124.54	14.12		201.29	19.26		677.97	60.52	
> 4 people	115	143.04	22.31		273.96	46.33		252.71	54.65		259.93	51.73		102.35	13.87		181.08	31.05		627.11	102.99	
Household has children under age 18?	826																					
Yes child < 18	397	127.25	14.68	0.0116	266.58	25.20	0.3938	248.92	30.12	0.8313	244.58	24.23	0.9222	116.57	10.23	0.5695	189.66	16.68	0.4936	615.38	51.71	0.3007
No child <18	429	174.30	18.45		290.28	22.70		243.54	16.06		247.43	23.88		108.85	11.39		199	12.72		669.38	38.30	
Household Race	814																					
White	622	147.17	15.95		253.94	20.99		232.86	20.59		261.31	23.92		111.70	10.42		190.91	14.59		614.46	41.02	
Black	116	201.82	29.69	0.1834	472.31	66.52	0.0010	387.17	65.75	0.0328	161.43	26.24	0.0563	137.20	11.71	0.0681	227.78	22.73	0.0672	830.46	105.87	0.1260
Other	76	151.48	33.90		386.18	69.39		201.87	55.44		222.99	42.12		83.46	18.24		189.77	27.83		730.99	96.35	
Household Ethnicity	820																					
Hispanic	85	196.82	36.96	0.2243	390.48	86.14	0.1117	248.94	66.16	0.9016	270.78	76.18	0.6948	108.14	17.43	0.8425	219.57	35.65	0.2356	865.82	124.94	0.0336
Non-hispanic	735	150.41	15.12		269.09	19.80		241.06	17.20		241.46	19.24		112.18	9.73		191.99	13.05		624.75	37.04	

Factor	Total	Bedroom Bed			Bedroom Floor			Living Room Floor			Living Room Upholstery			Kitchen Floor			Geometric mean			Maximum		
	Sample Size **	Geometric Mean	Geometric mean SE	p-values	Geometric Mean	Geometric mean SE	p-values	Geometric Mean	Geometric mean SE	p-values	Geometric Mean	Geometric mean SE	p-values	Geometric Mean	Geometric mean SE	p-values	Geometric Mean	Geometric mean SE	p-values	Geometric Mean	Geometric mean SE	p-values
Education	791																					
Less Than High School	71	148.14	27.77		289.17	67.80		335.17	97.77		331.08	51.65		94.54	23.56		214.45	36.05		752.36	132.94	
High School	176	220.41	21.14	<.0001	306.72	43.78	0.5003	320.90	44.71	0.0190	345.09	38.56	0.0003	124.42	20.64	0.4958	234.65	23.76	0.0115	759.88	80.67	0.0242
College	544	133.02	14.97		256.36	18.34		209.80	15.14		209.26	19.24		104.63	8.32		175.01	10.43		578.55	27.75	
Does anyone in the home smoke cigarettes?	825																					
Yes	332	186.25	18.66	0.0220	331.98	31.88	0.0131	323.03	33.40	<.0001	280.35	32.72	0.0548	125.74	12.72	0.0980	233.83	17.92	<.0001	790.54	59.57	0.0003
No	493	135.58	16.35		249.83	20.56		205.55	17.07		228.88	16.83		103.07	10.07		173.41	12.63		567.71	37.3	
Last time kitchen floor/carpet was cleaned?	810																					
< One Week	503	160.83	18.88	0.2343	270.54	27.28	0.9209	215.7	23.91	0.0623	243.16	23.13	0.7905	106.47	9.96	0.3896	187.82	15.63	0.6518	654.43	46.17	0.4118
One week or >	307	135.81	13.95		274.35	23.25		282.34	23.96		236.06	21.38		117.28	11.52		196.96	14.02		604.87	43.97	
Last time living room floor/carpet was cleaned?	797																					
< One Week	484	149.22	19.63	0.9910	254.15	24.91	0.3904	213.82	18.39	0.0101	233.42	20.28	0.4606	101.78	8.10	0.2052	176.77	13.48	0.1302	612.52	43.26	0.4743
One week or >	313	149.47	11.23		289.53	28.61		281.46	27.64		252.05	24.38		117.41	12.2		207.6	13.84		652.45	42.79	
Last time bedroom floor/carpet was cleaned?	771																					
< One Week	396	154.73	20.00	0.4872	244.79	24.96	0.1938	213.19	20.10	0.0834	234.23	21.05	0.8225	106.04	8.75	0.5534	177.54	14.26	0.4355	614.15	47.56	0.6690
One week or >	375	141.27	12.89		292.89	26.82		264.59	27.68		240.24	24.3		113.88	13.13		199.44	16.36		641.62	48.45	

** Actual sample size of available dust weight at each location may be less.

Table S2. Factors entered into linear regression models predicting dust weight for each location.

Bedroom Dust Weight (mg) Summarized Levels of Factors

Factor		Total Sample Size **	Bedroom Bed Geometric Mean	Geometric mean SE	p-values
Household Income by 20K		723			
	$0-19,999	169	192.77	20.49	0.0008
	$20,000-39,999	219	187.98	19.45	
	$40,000-59,999	143	126.874	34.52	
	$60,000+	192	92.38	11.98	
Season		784			
	Winter	189	155.51	35.49	0.4515
	Summer	276	128.94	32.01	
	Fall	319	182.46	21.86	
Census Region		781			
	Northeast	148	154.74	22.86	0.9355
	Midwest	184	165.97	27.05	
	South	265	144.34	30.63	
	West	184	153.97	11.64	
Household Construction Year		781			
	1978-1998	210	116.99	18.87	0.0334
	1960-1977	254	124.57	12.97	
	1946-1959	131	182.20	30.73	
	1940-1945	41	243.82	57.42	
	1939 or earlier	145	257.80	37.92	
How many stories, including basement?		778			
	1 story	314	125.74	25.12	0.3827
	2-3 stories	412	170.21	14.58	
	4 or more stories	52	208.83	25.79	
Main heating source in home?		778			
	Electric	219	148.07	18.02	0.9754
	Gas	407	149.38	19.35	
	Other/none	152	174.72	27.51	

Air conditioning system in home?		780			
	Air conditioning	620	144.09	15.38	0.0502
	No air conditioning	160	201.99	31.09	
Any pets currently in house?		773			
	Yes	378	142.10	18.55	0.2403
	No	395	166.85	17.98	
Air filtration device in home?		764			
	Yes	94	165.84	25.11	0.3186
	No	670	150.57	15.06	
Dehumidifies used in home?		765			
	Yes	128	191.03	31.54	0.2008
	No	637	152.70	15.75	
How many people live in house?		781			
	1 person	109	200.01	29.81	
	2 people	236	176.69	27.50	
	3 people	162	106.29	14.51	0.0019
	4 people	161	138.48	22.40	
	> 4 people	113	143.04	22.31	
Does anyone in the home smoke cigarettes?		777			
	Yes	312	186.25	18.66	0.2071
	No	465	135.58	16.35	
Household Race		766			
	White	588	147.17	15.95	
	Black	108	201.82	29.69	0.0554
	Other	70	151.48	33.90	
Household Ethnicity		773			
	Hispanic	81	196.82	36.96	0.7866
	Non-hispanic	692	150.41	15.12	
Education		745			
	Less Than High School	65	148.14	27.77	
	High School	165	220.41	21.14	0.0147
	College	515	133.02	14.97	

Urbanization		781			
	MSA >= 1 million	250	154.48	11.27	0.9818
	MSA < 1 million	400	155.01	16.03	
	Non-MSA PSU	131	147.83	45.50	
Household has children under age 18?		778			
	Yes child < 18	386	127.25	14.68	0.0837
	No child <18	392	174.30	18.45	
Bedroom Floor		749			
	Carpeted	565	143.34	13.4	0.0038
	Smooth and Cleanable	174	168.53	26.15	
	Not Smooth and Cleanable	10	585.56	246.33	
Bedroom Mat		765			
	No	747	151.02	13.87	0.1191
	Yes	18	257.59	97.19	
Bedroom Area Rug		765			
	No	690	149.13	15.15	0.1963
	Yes	75	194.68	42.59	
Bedroom Wall-to-Wall Carpet		765			
	No	189	195.75	25.58	0.0167
	Yes	576	142.43	14.57	
No Carpet in Bedroom		765			
	No	647	146.59	14.53	0.0399
	Yes	118	204.61	30.22	
Observed impremeable pillow or mattres covers?		757			
	Yes	194	69.31	16.3	0.0002
	No	563	194.73	16.94	
Use impermeable pillow or mattress covers?		773			
	Yes	30	138.74	38.97	0.9023
	No	743	153.15	14.57	
Last time bedroom floor/carpet was cleaned?		737			
	< One Week	377	154.73	20.00	0.7451
	One week or >	360	141.27	12.89	

Bedding washed within last week		729			
	Yes	523	144.61	19.55	0.3398
	No	206	179.17	18.59	
Water temperature when washing bedding		736			
	Hot	249	193.18	24.66	
	Warm	348	139.15	15.82	0.0852
	Cold	139	136.56	18.62	
Temperature in Bedroom (F)		763			
	< 65	30	171.52	40.11	
	65 - 74	294	169.77	18.59	0.7097
	75 - 84	389	148.05	17.59	
	> = 84	50	97.01	68.46	
Humidity in Bedroom (%)		768			
	< 40	136	174.73	68.3	
	40 - 49	247	163.89	18.64	
	50 - 59	176	168.12	19.74	0.3677
	60 - 69	143	126.55	15.25	
	> = 70	66	97.47	23.84	
Observed Moisture in the Bedroom		774			
	Yes	59	375.72	98.25	0.0028
	No	715	142.65	14.05	
Stuffed Animals found on the bed		756			
	Yes	190	103.79	12.21	0.0001
	No	566	176.02	17.25	
Carpet In the Bedroom		765			
	Yes	644	146.67	14.55	0.0446
	No	121	202.23	29.58	

** Actual sample size of available dust weight at each location may be less.

Bedroom Floor Dust Weight (mg) Summarized by Levels of Factors

Factor		Total	Bedroom Floor		
		Sample Size **	Geometric Mean	Geometric mean SE	p-values
Household Income by 20K		725			
	$0-19,999	168	430.73	57.72	<.0001
	$20,000-39,999	217	348.93	38.09	
	$40,000-59,999	146	257.34	26.92	
	$60,000+	194	151.60	25.12	
Season		784			
	Winter	188	300.64	55.49	0.1221
	Summer	276	222.94	28.61	
	Fall	320	335.65	38.62	
Census Region		784			
	Northeast	148	219.86	38.38	0.0510
	Midwest	184	228.11	26.77	
	South	269	327.91	42.54	
	West	183	334.33	44.23	
Household Construction Year		784			
	1978-1998	213	230.09	27.79	0.0070
	1960-1977	255	257.69	29.68	
	1946-1959	131	270.85	48.85	
	1940-1945	41	418.49	70.00	
	1939 or earlier	144	405.37	62.40	
How many stories, including basement?		781			
	1 story	311	308.88	40.89	0.5494
	2-3 stories	418	263.04	25.87	
	4 or more stories	52	254.41	40.62	
Main heating source in home?		781			
	Electric	220	318.03	29.11	0.3496
	Gas	408	261.62	30.33	
	Other/none	153	275.48	43.92	
Does home have air conditioning?		783			
	Air conditioning	624	261.05	19.51	0.0523
	No air conditioning	159	379.62	65.63	
Any pets currently in house?		776			
	Yes	382	286.40	22.01	0.7295
	No	394	276.61	26.39	

Air filtration device in home?		766			
	Yes	98	244.56	34.81	0.3633
	No	668	283.85	22.09	
Dehumidifies used in home?		768			
	Yes	127	203.75	24.22	0.0172
	No	641	299.00	25.49	
How many people live in house?		784			
	1 person	110	326.65	56.18	
	2 people	240	269.61	28.02	
	3 people	164	266.12	34.17	0.8605
	4 people	159	273.52	34.15	
	> 4 people	111	273.96	46.33	
Does anyone in the home smoke cigarettes?		780			
	Yes	313	331.98	31.88	0.0131
	No	467	249.83	20.56	
Household Race		770			
	White	590	253.94	20.99	
	Black	108	472.31	66.52	0.0010
	Other	72	386.18	69.39	
Household Ethnicity		776			
	Hispanic	80	390.48	86.14	0.1117
	Non-hispanic	696	269.09	19.80	

Education		748			
	Less Than High School	63	289.17	67.80	0.5003
	High School	167	306.72	43.78	
	College	518	256.36	18.34	
Urbanization		784			
	MSA >= 1 million	256	233.52	24.23	0.1260
	MSA < 1 million	400	272.78	24.99	
	Non-MSA PSU	128	356.60	63.95	
Household has children under age 18?		781			
	Yes child < 18	382	266.58	25.20	0.3938
	No child <18	399	290.28	22.70	
Bedroom Floor		753			
	Carpeted	568	314.13	23.61	0.0012
	Smooth and Cleanable	175	176.62	26.94	
	Not Smooth and Cleanable	10	494.01	231.49	
Bedroom Mat		769			
	No	751	277.06	19.38	0.4120
	Yes	18	390.10	166.72	
Bedroom Area Rug		769			
	No	693	267.85	19.85	0.0140
	Yes	76	409.75	68.77	
Bedroom Wall-to-Wall Carpet		769			
	No	187	210.60	27.84	0.0191
	Yes	582	303.52	25.34	
No Carpet in Bedroom		769			
	No	654	310.41	24.82	0.0001
	Yes	115	137.04	24.13	
Last time bedroom floor/carpet was cleaned?		741			
	< One Week	383	244.79	24.96	0.1938
	One week or >	358	292.89	26.82	

Temperature in Bedroom (F)		768			
	< 65	31	334.38	66.31	0.8075
	65 - 74	295	277.60	28.03	
	75 - 84	391	274.26	26.79	
	> = 84	51	284.46	65.83	
Humidity in Bedroom (%)		772			
	< 40	138	452.49	84	0.0157
	40 - 49	246	278.29	24.94	
	50 - 59	174	252.14	33.31	
	60 - 69	147	203.24	23.56	
	> = 70	67	245.14	35	
Observed Moisture in the Bedroom		777			
	Yes	55	583.07	151.34	0.0033
	No	722	266.03	18.03	
Carpet In the Bedroom		769			
	Yes	651	310.50	24.83	<.0001
	No	118	139.52	23.27	

** Actual sample size of available dust weight at each location may be less.

Kitchen Floor Dust Weight (mg) Summarized by Levels of Factors

Factor		Total	Kitchen Floor		
		Sample Size **	Geometric Mean	Geometric mean SE	p-values
Household Income by 20K		736			
	$0-19,999	175	149.52	18.77	0.0249
	$20,000-39,999	218	99.98	15.13	
	$40,000-59,999	149	129.48	22.68	
	$60,000+	194	85.21	12.78	
Season		794			
	Winter	189	124.57	25.34	0.3697
	Summer	275	93.01	12.74	
	Fall	330	122.35	17.61	
Census Region		794			
	Northeast	148	107.14	20.03	0.8854
	Midwest	189	126.48	19.84	
	South	268	108.25	14.65	
	West	189	103.61	15.56	
Household Construction Year		794			
	1978-1998	210	92.66	11.15	<.0001
	1960-1977	254	82.91	8.50	
	1946-1959	132	121.23	19.75	
	1940-1945	44	235.90	49.32	
	1939 or earlier	154	172.04	21.96	
How many stories, including basement?		790			
	1 story	315	113.34	15.39	0.7307
	2-3 stories	417	108.46	12.23	
	4 or more stories	58	121.01	21.66	

Main heating source in home?		791			
	Electric	216	99.49	9.25	
	Gas	420	110.12	13.49	0.4541
	Other/none	155	128.20	23.98	
Does home have air conditioning?		793			
	Air conditioning	626	105.52	8.76	0.1345
	No air conditioning	167	143.14	27.09	
Any pets currently in house?		786			
	Yes	384	124.76	12.74	0.0345
	No	402	99.97	9.65	
Air filtration device in home?		775			
	Yes	93	127.72	23.67	0.2221
	No	682	107.66	8.54	
Dehumidifies used in home?		779			
	Yes	125	114.10	16.03	0.8697
	No	654	110.92	9.74	
How many people live in house?		794			
	1 person	116	134.19	23.46	
	2 people	242	100.18	13.49	
	3 people	162	106.60	12.41	0.5486
	4 people	162	124.54	14.12	
	> 4 people	112	102.35	13.87	
Does anyone in the home smoke cigarettes?		790			
	Yes	319	125.74	12.72	0.4195
	No	471	103.07	10.07	
Household Race		780			
	White	597	111.70	10.42	
	Black	110	137.20	11.71	0.0255
	Other	73	83.46	18.24	
Household Ethnicity		785			
	Hispanic	83	108.14	17.43	0.2629
	Non-hispanic	702	112.18	9.73	

Education		756			
	Less Than High School	66	94.54	23.56	
	High School	171	124.42	20.64	0.2529
	College	519	104.63	8.32	
Urbanization		794			
	MSA >= 1 million	266	92.38	10.18	
	MSA < 1 million	395	97.79	10.65	0.0029
	Non-MSA PSU	133	175.48	30.91	
Household has children under age 18?		791			
	Yes child < 18	383	116.57	10.23	0.5856
	No child <18	408	108.85	11.39	
Kitchen Floor		770			
	Carpeted	56	349.94	65.84	
	Smooth and Cleanable	686	99.68	7.14	<.0001
	Not Smooth and Cleanable	28	127.19	59.77	
Kitchen Mat		781			
	No	688	106.02	8.45	0.2142
	Yes	93	154.06	26.68	
Kitchen Area Rug		781			
	No	714	104.28	8.22	0.0005
	Yes	67	207.08	41.25	
Kitchen Wall-to-Wall Carpet		781			
	No	739	102.22	7.08	<.0001
	Yes	42	415.12	75.34	
No Carpet in Kitchen		781			
	No	186	192.97	24.58	<.0001
	Yes	595	92.08	6.76	
Last time kitchen floor/carpet was cleaned?		778			
	< One Week	482	106.47	9.96	0.3101
	One week or >	296	117.28	11.52	

Temperature in Kitchen (F)		785			
	< 65	62	146.84	36.27	0.9527
	65 - 74	302	108.12	14.58	
	75 - 84	354	111.61	11.94	
	> = 84	67	101.13	21.59	
Humidity in Kitchen (%)		789			
	< 40	166	160.39	22.94	0.0072
	40 - 49	236	108.33	11.78	
	50 - 59	191	104.81	13.57	
	60 - 69	123	84.43	12.73	
	> = 70	73	101.67	24.74	
Observed Moisture in the Kitchen		793			
	Yes	156	218.84	46.13	0.0012
	No	637	95.41	7.48	
Carpet In the Kitchen		781			
	Yes	191	194.13	24.04	<.0001
	No	590	91.31	6.68	

** Actual sample size of available dust weight at each location may be less.

Living Room Floor Dust Weight (mg) Summarized by Levels of Factors

Factor		Total	Living Room Floor		
		Sample Size **	Geometric Mean	Geometric mean SE	p-values
Household Income by 20K					
	$0-19,999	175	383.22	60.17	<.0001
	$20,000-39,999	216	317.04	29.69	
	$40,000-59,999	141	211.13	27.83	
	$60,000+	189	123.17	19.81	
Season					
	Winter	181	245.73	47.16	0.2332
	Summer	275	198.95	32.55	
	Fall	323	301.12	34.08	
Census Region					
	Northeast	146	148.76	27.39	0.0805
	Midwest	189	280.76	30.88	
	South	257	282.85	33.77	
	West	187	261.21	55.32	
Household Construction Year					
	1978-1998	201	213.87	21.64	0.0674
	1960-1977	253	211.40	23.63	
	1946-1959	134	274.74	51.02	
	1940-1945	43	372.71	100.41	
	1939 or earlier	148	300.26	50.95	
How many stories, including basement?					
	1 story	307	280.14	37.76	0.2757
	2-3 stories	409	227.39	22.47	
	4 or more stories	60	205.85	45.65	

Main heating source in home?					
	Electric	217	254.71	28.68	
	Gas	405	242.37	29.28	0.7426
	Other/none	154	234.10	37.84	
Does home have air conditioning?					
	Air conditioning	614	229.86	18.21	0.0260
	No air conditioning	164	317.27	50.31	
Any pets currently in house?					
	Yes	371	289.21	30.71	0.0091
	No	400	214.42	18.33	
Air filtration device in home?					
	Yes	97	173.43	29.41	0.0958
	No	664	254.29	19.97	
Dehumidifies used in home?					
	Yes	123	175.32	23.15	0.0439
	No	643	262.54	21.67	
How many people live in house?					
	1 person	119	262.00	42.88	
	2 people	239	242.47	21.70	
	3 people	158	216.48	24.87	0.7018
	4 people	156	258.63	36.53	
	> 4 people	107	252.71	54.65	
Does anyone in the home smoke cigarettes?					
	Yes	311	323.03	33.40	0.0073
	No	464	205.55	17.07	
Household Race					
	White	584	232.86	20.59	
	Black	110	387.17	65.75	0.1142
	Other	70	201.87	55.44	
Household Ethnicity					
	Hispanic	78	248.94	66.16	0.7029
	Non-hispanic	693	241.06	17.20	

Education					
	Less Than High School	511	209.80	15.14	0.0240
	High School	167	320.90	44.71	
	College	64	335.17	97.77	
Urbanization					
	MSA >= 1 million	259	215.29	22.15	0.1388
	MSA < 1 million	389	229.19	21.71	
	Non-MSA PSU	131	320.76	67.29	
Household has children under age 18?					
	Yes child < 18	367	248.92	30.12	0.7678
	No child <18	409	243.54	16.06	
Living Room Floor					
	Carpeted	572	284.9	21.86	0.0018
	Smooth and Cleanable	176	139.39	20.86	
	Not Smooth and Cleanable	5	167.44	121.58	
Living Room Mat					
	No	756	238.77	18.73	0.0015
	Yes	19	506.46	113.99	
Living Room Area Rug					
	No	642	239.24	21.1	0.3287
	Yes	133	264.55	41.27	
Living Room Wall-to-Wall Carpet					
	No	197	157.06	23.69	0.0029
	Yes	578	283.07	23.25	
No Carpet in Living Room					
	No	681	271.86	19.47	0.0002
	Yes	94	104.46	24.48	
Last time Living Room floor/carpet cleaned					
	< One Week	463	213.82	18.39	0.0120
	One week or >	299	281.46	27.64	

Last time Living Room Upholstery Cleaned					
	1-4 Weeks	18	205.82	72.92	0.1685
	One Month or More	175	294.41	37.42	
	Never	486	226.12	21.36	
Temperature in Living Room (F)					
	< 65	46	295.64	73.33	0.6006
	65 - 74	312	239.5	28.41	
	75 - 84	354	239.15	23.55	
	> = 84	59	270.45	67.74	
Humidity in Living Room (%)					
	< 40	164	294.49	69.8	0.6466
	40 - 49	232	241.42	29.93	
	50 - 59	194	254.05	23.66	
	60 - 69	119	197.05	20.81	
	> = 70	66	191.69	39.72	
Observed Moisture in the Living Room					
	Yes	48	553.28	177.99	0.0061
	No	721	232.5	17.51	
Carpet In the Living Room					
	Yes	681	272.51	19.54	0.0001
	No	94	103.05	24.03	

** Actual sample size of available dust weight at each location may be less.

Living Room Upholstery Dust Weight (mg) Summarized by Levels of Factors

Factor		Total	Living Room Upholstery		
		Sample Size **	Geometric Mean	Geometric mean SE	p-values
Household Income by 20K					
	$0-19,999	168	300.44	46.48	<0.0001
	$20,000-39,999	212	292.62	34.34	
	$40,000-59,999	142	247.20	28.17	
	$60,000+	182	140.42	16.72	
Season					
	Winter	173	258.97	43.94	0.5301
	Summer	273	222.74	34.28	
	Fall	312	261.76	34.30	
Census Region					
	Northeast	144	183.59	24.58	0.0269
	Midwest	182	326.08	57.96	
	South	252	228.85	32.79	
	West	180	267.62	40.70	
Household Construction Year					
	1978-1998	201	198.67	24.12	0.0211
	1960-1977	241	214.43	23.56	
	1946-1959	134	309.51	41.48	
	1940-1945	42	348.48	99.31	
	1939 or earlier	140	314.56	50.15	
How many stories, including basement?					
	1 story	302	257.21	32.28	0.6141
	2-3 stories	394	238.54	23.76	
	4 or more stories	60	233.38	49.59	

Main heating source in home?					
	Electric	213	222.25	31.21	
	Gas	391	255.13	28.91	0.5626
	Other/none	151	256.49	34.46	
Does home have air conditioning?					
	Air conditioning	598	238.58	21.03	0.5660
	No air conditioning	159	275.52	39.48	
Any pets currently in house?					
	Yes	363	266.58	27.17	0.1646
	No	387	228.81	20.65	
Air filtration device in home?					
	Yes	92	222.96	34.45	0.5993
	No	648	246.37	20.49	
Dehumidifies used in home?					
	Yes	121	310.83	62.52	0.0943
	No	624	234.58	17.53	
How many people live in house?					
	1 person	112	284.28	38.76	
	2 people	231	246.25	33.00	
	3 people	158	187.48	17.12	0.1641
	4 people	158	275.43	34.25	
	> 4 people	105	259.93	51.73	
Does anyone in the home smoke cigarettes?					
	Yes	305	280.35	32.72	0.0505
	No	449	228.88	16.83	
Household Race					
	White	574	261.31	23.92	
	Black	103	161.43	26.24	0.0066
	Other	66	222.99	42.12	
Household Ethnicity					
	Hispanic	72	270.78	76.18	0.6654
	Non-hispanic	678	241.46	19.24	
Education					
	Less Than High School	61	331.08	51.65	
	High School	165	345.09	38.56	0.0001
	College	495	209.26	19.24	

Urbanization					
	MSA >= 1 million	250	194.43	20.13	0.0300
	MSA < 1 million	381	237.36	26.12	
	Non-MSA PSU	127	341.47	68.58	
Household has children under age 18?					
	Yes child < 18	361	244.58	24.23	0.5881
	No child <18	394	247.43	23.88	
Living Room Floor					
	Carpeted	561	250.8	22.41	0.2230
	Smooth and Cleanable	169	239.87	39.3	
	Not Smooth and Cleanable	5	115.16	46.3	
Living Room Mat					
	No	735	245.1	20.22	0.8834
	Yes	19	230.59	63.12	
Living Room Area Rug					
	No	622	247.74	20.71	0.6346
	Yes	132	231.19	41.49	
Wall-to-Wall Carpet in Living Room					
	No	189	235.09	42.03	0.6857
	Yes	565	247.93	18.97	
No Carpet in Living Room					
	No	667	240.88	17.82	0.8617
	Yes	87	280.28	88.41	
Last time Living Room floor/carpet was cleaned?					
	< One Week	452	233.42	20.28	0.3525
	One week or >	288	252.05	24.38	
Last time Living Room Upholstery cleaned?					
	1-4 Weeks	18	149.93	81.42	0.0010
	One Month or More	173	394.2	39.33	
	Never	482	218.56	20.56	
Temperature in Living Room (F)					
	< 65	44	273.05	63.86	0.0838
	65 - 74	297	254.23	32.45	
	75 - 84	352	253.49	25.45	
	> = 84	58	137.06	37.78	

Humidity in Living Room (%)					
	< 40	161	294.58	60.71	0.1656
	40 - 49	222	245.46	25.08	
	50 - 59	190	251.85	30.69	
	60 - 69	115	247.21	27.25	
	> = 70	66	121.09	29.59	
Observed Moisture in the Living Room					
	Yes	47	244.3	53.98	0.6801
	No	701	248.47	20.53	
Carpet In the Living Room					
	Yes	667	241.39	17.88	0.8280
	No	87	275.06	86.94	

Table S3. Coefficients from linear regression models.

SITE/FACTOR		Logged Coefficient of Level of Effect	**True Coefficient of Level of Effect**
**Bedroom Bed**			
Intercept		2.56	**363.08**
Household Income by 20k			
	$0-19,999	0.29	**1.95**
	$20,000-39999	0.31	**2.06**
	$40,000-59999	0.17	**1.47**
	60,000 +	0.00	**1.00**
Census Region			
	Northeast	-0.29	**0.52**
	Midwest	-0.26	**0.56**
	South	-0.10	**0.80**
	West	0.00	**1.00**
HU Construction Year			
	1978-1998	-0.21	**0.61**
	1960-1977	-0.21	**0.61**
	1946-1959	-0.05	**0.89**
	1940-1945	0.05	**1.11**
	1939 or earlier	0.00	**1.00**
How many stories, including basement?			
	1 story	-0.34	**0.46**
	2 to 3 stories	-0.11	**0.78**
	4 or more stories	0.00	**1.00**
Observed Protective Mattress Covers?			
	No	-0.43	**0.37**
	Yes	0.00	**1.00**
Observed Moisture in the Bedroom?			
	No	0.28	**1.92**
	Yes	0.00	**1.00**
Stuffed Animals On Bed?			
	No	-0.16	**0.70**
	Yes	0.00	**1.00**
**Bedroom Floor**			
Intercept		2.24	**172.19**
Household Income by 20k			
	$0-19,999	0.39	**2.43**

	$20,000-39999	0.32	**2.07**
	$40,000-59999	0.16	**1.46**
	60,000 +	0.00	**1.00**
Census Region			
	Northeast	-0.16	**0.70**
	Midwest	-0.10	**0.80**
	South	0.07	**1.19**
	West	0.00	**1.00**
HU Construction Year			
	1978-1998	-0.29	**0.51**
	1960-1977	-0.31	**0.49**
	1946-1959	-0.20	**0.63**
	1940-1945	0.07	**1.17**
	1939 or earlier	0.00	**1.00**
Does home have air conditioning			
	Air conditioning	-0.19	**0.65**
	No air conditioning	0.00	**1.00**
Household: Any pets?			
	Pet(s)	0.10	**1.26**
	No Pet(s)	0.00	**1.00**
Race			
	White	-0.20	**0.63**
	Black	0.09	**1.22**
	Other	0.00	**1.00**
Last time bedroom floor was cleaned			
	1	-0.13	**0.75**
	2	0.00	**1.00**
Humidity level in the bedroom			
	1	0.24	**1.74**
	2	0.11	**1.29**
	3	0.03	**1.06**
	4	-0.04	**0.91**
	5	0.00	**1.00**
OBMOISTB			
	1	0.21	**1.61**
	2	0.00	**1.00**
DICARPET			
	1	0.54	**3.44**
	2	0.00	**1.00**

Kitchen Floor

Intercept		2.18	**149.62**
HU Construction Year			
	1978-1998	-0.25	**0.57**
	1960-1977	-0.27	**0.54**

1946-1959	-0.12	**0.76**
1940-1945	0.10	**1.25**
1939 or earlier	0.00	**1.00**
Household: Any pets?		
Pet(s)	0.15	**1.40**
No Pet(s)	0.00	**1.00**
Race		
White	-0.01	**0.98**
Black	0.16	**1.46**
Other	0.00	**1.00**
Urbanization		
MSA >= 1 million	-0.35	**0.45**
MSA < 1 million	-0.17	**0.67**
Non-MSA PSU	0.00	**1.00**
Humidity Level in the Kitchen		
1	0.13	**1.34**
2	-0.02	**0.95**
3	-0.06	**0.86**
4	-0.17	**0.67**
5	0.00	**1.00**
Observed Moisture in the Kitchen?		
Yes	0.28	**1.88**
No	0.00	**1.00**
Any carpet present?		
Yes	0.32	**2.08**
No	0.00	**1.00**

Living Room Floor

Intercept	1.80	**63.68**
Household Income by 20k		
$0-19,999	0.53	**3.40**
$20,000-39999	0.42	**2.61**
$40,000-59999	0.21	**1.61**
60,000 +	0.00	**1.00**
Census Region		
Northeast	-0.25	**0.56**
Midwest	0.06	**1.15**
South	0.13	**1.35**
West	0.00	**1.00**
HU Construction Year		
1978-1998	-0.18	**0.66**
1960-1977	-0.27	**0.53**
1946-1959	-0.11	**0.77**
1940-1945	0.13	**1.35**
1939 or earlier	0.00	**1.00**

Does home have air conditioning			
	Air conditioning	-0.24	**0.58**
	No air conditioning	0.00	**1.00**
Household: Any pets?			
	Pet(s)	0.29	**1.95**
	No Pet(s)	0.00	**1.00**
Last Time Living Room Floor Cleaned			
	1	-0.17	**0.67**
	2	0.00	**1.00**
Any carpet present?			
	1	0.61	**4.07**
	2	0.00	**1.00**

Living Room Upholstery

Intercept		0.00	**1.00**
How many people in the home?			
	1 Person	-0.18	**0.66**
	2 Persons	-0.25	**0.57**
	3 Persons	-0.11	**0.77**
	4 Persons	0.05	**1.13**
	More Than 4	0.00	**1.00**
Race			
	White	0.07	**1.19**
	Black	-0.30	**0.50**
	Other	0.00	**1.00**
Education			
	College	0.04	**1.10**
	High School	0.26	**1.83**
	< High School	0.00	**1.00**
Last Time living room upholstery cleaned?			
	1	-0.18	**0.67**
	2	0.22	**1.65**
	3	0.00	**1.00**
Temperature in the Living Room			
	1	0.30	**1.97**
	2	0.45	**2.81**
	3	0.41	**2.54**
	4	0.00	**1.00**

Table S4. Adjusted odds ratios[1] (OR) for current asthma and wheeze, for dust weight (dichotomized at the median value), by house indices, from the National Survey of Lead and Allergens in Housing, 1998-1999. Stratified by allergy status (doctor-diagnosed allergies).

Locations	Allergies=No<br>Asthma (n=41)<br>Adjusted OR<br>(95% CI)	Allergies=Yes<br>Asthma (n=130)<br>Adjusted OR<br>(95% CI)	p-value[2]	Allergies=No<br>Wheeze (n=167)<br>Adjusted OR<br>(95% CI)	Allergies=Yes<br>Wheeze (n=176)<br>Adjusted OR<br>(95% CI)	p-value
Mean Index						
< Median	Ref	Ref		Ref	Ref	
> Median	2.35 (0.98, 5.64)	1.20 (0.74, 1.93)	0.16	1.92 (1.11, 3.31)	1.46 (0.93, 2.27)	0.45
Max. Index						
< Median	Ref	Ref		Ref	Ref	
> Median	1.32 (0.56, 3.10)	1.25 (0.68, 2.30)	0.67	1.29 (0.71, 2.35)	1.80 (0.95, 3.40)	0.41

[1] Adjusted for sex, age (categorized in decades), race, education, and environmental tobacco smoke exposure
[2] p-value for interaction between dust weight and allergy status

Table S5. Adjusted odds ratios[1] (OR) for current asthma and wheeze, for dust weight (dichotomized at the median value), by house indices, from the National Survey of Lead and Allergens in Housing, 1998-1999. Stratified by urban residence status.

Locations	Urban=No<br>Asthma (n=114)<br>Adjusted OR (95% CI)	Urban=Yes<br>Asthma (n=60)<br>Adjusted OR (95% CI)	p-value[2]	Urban=No<br>Wheeze (n=250)<br>Adjusted OR (95% CI)	Urban=Yes<br>Wheeze (n=103)<br>Adjusted OR (95% CI)	p-value
Mean Index						
< Median	Ref	Ref		Ref	Ref	
> Median	2.01 (1.20, 3.35)	0.98 (0.51, 1.89)	0.12	1.84 (1.27, 2.68)	1.73 (088, 3.39)	0.83
Max. Index						
< Median	Ref	Ref	0.56	Ref	Ref	0.05
> Median	1.73 (0.86, 3.48)	1.16 (0.65, 2.07)		1.38 (0.93, 2.06)	2.79 (1.69, 4.61	

[1] Adjusted for sex, age (categorized in decades), race, education, and environmental tobacco smoke exposure
[2] p-value for interaction between dust weight and urban residence status

Table S6. Logistic regression models, with adjustment for allergen and endotoxin concentrations.

ASTHMA

Referent = Quartile 1					Adjusted Model Plus:			
	**Adjusted Model**	**Alternaria**	**Bla G 1**	**Can f 1**	**Der f + Der p**	**Endotoxin**	**Fel d 1**	**Mus m 1**
Bed Q2	1.23 (0.63, 2.40)	1.30 (0.68, 2.49)	0.81 (0.36, 1.80)	1.26 (0.63, 2.51)	1.28 (0.65, 2.50)	1.24 (0.63, 2.44)	1.18 (0.61, 2.30)	1.07 (0.53, 2.18)
Bed Q3	1.42 (0.72, 2.78)	1.56 (0.79, 3.10)	1.67 (0.72, 3.86)	1.46 (0.74, 2.85)	1.39 (0.71, 2.71)	1.42 (0.71, 2.81)	1.37 (0.70, 2.68)	1.30 (0.66, 2.55)
Bed Q4	1.89 (1.09, 3.27)	2.04 (1.15, 3.59)	1.98 (1.10, 3.57)	1.90 (1.09, 3.31)	1.87 (1.09, 3.22)	1.97 (1.09, 3.57)	1.81 (1.06, 3.10)	1.72 (0.99, 2.97)
BR Floor Q2	0.83 (0.41, 1.68)	0.86 (0.42, 1.73)	1.44 (0.66, 3.13)	0.78 (0.37, 1.63)	0.81 (0.41, 1.63)	0.73 (0.37, 1.45)	0.81 (0.40, 1.62)	1.07 (0.52, 2.22)
BR Floor Q3	0.88 (0.43, 1.80)	0.88 (0.43, 1.81)	1.23 (0.57, 2.65)	0.80 (0.39, 1.66)	0.87 (0.43, 1.76)	0.82 (0.40, 1.69)	0.85 (0.41, 1.75)	1.22 (0.59, 2.51)
BR Floor Q4	0.92 (0.53, 1.57)	0.90 (0.51, 1.56)	1.55 (0.80, 2.99)	0.86 (0.49, 1.49)	0.88 (0.51, 1.52)	0.85 (0.49, 1.46)	0.88 (0.51, 1.51)	1.09 (0.59, 2.00)
Kit Floor Q2	0.97 (0.49, 1.92)	0.97 (0.48, 1.94)	0.84 (0.43, 1.62)	0.95 (0.48, 1.90)	0.95 (0.48, 1.91)	0.94 (0.47, 1.91)	0.93 (0.47, 1.87)	1.03 (0.49, 2.17)
Kit Floor Q3	1.28 (0.64, 2.54)	1.24 (0.62, 2.50)	1.20 (0.57, 2.52)	1.23 (0.62, 2.45)	1.27 (0.63, 2.54)	1.16 (0.58, 2.33)	1.24 (0.63, 2.46)	1.03 (0.48, 2.18)
Kit Floor Q4	1.92 (1.03, 3.60)	1.81 (0.95, 3.44)	2.00 (1.15, 3.49)	1.81 (0.95, 3.46)	1.92 (1.01, 3.64)	1.75 (0.93, 3.31)	1.86 (1.00, 3.45)	1.54 (0.80, 2.94)
LR Floor Q2	0.65 (0.30, 1.42)	0.60 (0.28, 1.31)	0.64 (0.27, 1.50)	0.66 (0.30, 1.43)	0.64 (0.30, 1.39)	0.56 (0.25, 1.23)	0.63 (0.29, 1.38)	0.81 (0.36, 1.81)
LR Floor Q3	0.83 (0.44, 1.59)	0.79 (0.42, 1.51)	0.69 (0.33, 1.46)	0.79 (0.41, 1.53)	0.81 (0.42, 1.56)	0.74 (0.39, 1.41)	0.81 (0.42, 1.56)	1.20 (0.56, 2.60)
LR Floor Q4	1.52 (1.01, 2.29)	1.36 (0.86, 2.14)	2.31 (1.30, 4.12)	1.35 (0.88, 2.10)	1.52 (1.00, 2.30)	1.32 (0.87, 1.99)	1.48 (0.98, 2.24)	1.89 (1.08, 3.32)
LR Sofa Q2	1.30 (0.69, 2.45)	1.23 (0.65, 2.34)	1.02 (0.49, 2.15)	1.19 (0.63, 2.26)	1.30 (0.68, 2.46)	1.14 (0.60, 2.17)	1.27 (0.67, 2.40)	1.24 (0.61, 2.53)
LR Sofa Q3	0.63 (0.30, 1.33)	0.59 (0.28, 1.25)	0.73 (0.32, 1.64)	0.58 (0.27, 1.24)	0.64 (0.31, 1.35)	0.56 (0.27, 1.18)	0.61 (0.29, 1.29)	0.50 (0.25, 1.00)
LR Sofa Q4	0.98 (0.59, 1.62)	0.93 (0.56, 1.53)	1.38 (0.84, 2.26)	0.88 (0.52, 1.48)	1.00 (0.59, 1.69)	0.88 (0.52, 1.47)	0.94 (0.57, 1.53)	1.07 (0.62, 1.84)
House Mean Q2	1.27 (0.52, 3.15)	1.24 (0.51, 3.04)	2.00 (0.67, 5.96)	1.23 (0.51, 2.99)	1.33 (0.55, 3.22)	1.15 (0.48, 2.77)	1.23 (0.49, 3.08)	1.61 (0.60, 4.37)
House Mean Q3	1.58 (0.77, 3.28)	1.63 (0.77, 3.44)	2.69 (0.99, 7.33)	1.51 (0.73, 3.12)	1.54 (0.74, 3.19)	1.45 (0.69, 3.01)	1.53 (0.74, 3.17)	2.67 (1.24, 5.75)
House Mean Q4	1.79 (0.91, 3.51)	1.77 (0.89, 3.54)	4.22 (1.50, 11.9)	1.68 (0.84, 3.34)	1.78 (0.92, 3.47)	1.68 (0.86, 3.26)	1.72 (0.87, 3.40)	2.28 (1.01, 5.18)
House Max Q2	1.57 (0.81, 3.03)	1.60 (0.83, 3.08)	1.42 (0.61, 3.30)	1.50 (0.80, 2.84)	1.53 (0.80, 2.94)	1.57 (0.82, 3.02)	1.54 (0.80, 2.99)	2.46 (1.14, 5.29)
House Max Q3	1.93 (0.89, 4.18)	1.93 (0.88, 4.27)	2.86 (1.17, 6.98)	1.84 (0.84, 4.02)	1.80 (0.82, 3.93)	1.90 (0.86, 4.19)	1.90 (0.87, 4.14)	2.27 (1.05, 4.88)
House Max Q4	2.21 (1.08, 4.55)	2.14 (1.05, 4.38)	3.21 (1.09, 9.42)	2.04 (1.00, 4.17)	2.15 (1.06, 4.36)	2.18 (1.08, 4.39)	2.16 (1.06, 4.39)	2.67 (1.30, 5.50)

WHEEZE

**Comparison**	**Adjusted Model**	**Alternaria**	**Bla G 1**	**Can f 1**	**Der f + Der p**	**Endotoxin**	**Fel d 1**	**Mus m 1**
Bed Q2	1.42 (0.82, 2.47)	1.40 (0.82, 2.38)	0.75 (0.41, 1.38)	1.41 (0.82, 2.43)	1.44 (0.83, 2.51)	1.47 (0.85, 2.54)	1.37 (0.80, 2.35)	1.14 (0.71, 1.83)
Bed Q3	1.29 (0.79, 2.11)	1.28 (0.78, 2.10)	0.95 (0.55, 1.65)	1.26 (0.79, 2.01)	1.28 (0.79, 2.07)	1.24 (0.76, 2.03)	1.25 (0.76, 2.04)	1.21 (0.71, 2.09)
Bed Q4	1.99 (1.21, 3.28)	1.96 (1.17, 3.30)	1.98 (1.17, 3.36)	1.91 (1.19, 3.06)	1.98 (1.22, 3.22)	1.94 (1.16, 3.25)	1.92 (1.16, 3.16)	1.91 (1.07, 3.42)

BR Floor Q2	0.87 (0.40, 1.92)	0.85 (0.39, 1.83)	0.89 (0.49, 1.61)	0.83 (0.38, 1.82)	0.86 (0.39, 1.88)	0.76 (0.35, 1.68)	0.85 (0.39, 1.85)	0.74 (0.42, 1.28)
BR Floor Q3	0.79 (0.48, 1.29)	0.76 (0.46, 1.25)	1.21 (0.70, 2.07)	0.74 (0.45, 1.22)	0.78 (0.47, 1.28)	0.68 (0.42, 1.13)	0.76 (0.47, 1.24)	0.90 (0.56, 1.44)
BR Floor Q4	1.46 (0.86, 2.48)	1.40 (0.83, 2.37)	2.17 (1.28, 3.68)	1.36 (0.80, 2.33)	1.43 (0.85, 2.42)	1.27 (0.75, 2.16)	1.40 (0.84, 2.35)	1.59 (0.95, 2.65)
Kit Floor Q2	1.51 (0.91, 2.49)	1.44 (0.87, 2.41)	1.44 (0.72, 2.86)	1.48 (0.89, 2.46)	1.48 (0.90, 2.46)	1.48 (0.87, 2.50)	1.45 (0.87, 2.40)	1.21 (0.65, 2.24)
Kit Floor Q3	1.68 (0.99, 2.86)	1.61 (0.96, 2.71)	1.73 (0.95, 3.16)	1.59 (0.94, 2.71)	1.67 (0.98, 2.84)	1.57 (0.90, 2.76)	1.63 (0.97, 2.76)	1.42 (0.73, 2.74)
Kit Floor Q4	1.30 (0.78, 2.18)	1.23 (0.74, 2.05)	1.52 (0.94, 2.48)	1.24 (0.74, 2.10)	1.29 (0.77, 2.15)	1.22 (0.72, 2.07)	1.25 (0.74, 2.09)	1.20 (0.66, 2.18)
LR Floor Q2	1.19 (0.75, 1.89)	1.15 (0.71, 1.84)	1.24 (0.68, 2.25)	1.18 (0.73, 1.90)	1.18 (0.74, 1.87)	1.06 (0.64, 1.77)	1.15 (0.72, 1.82)	1.21 (0.74, 1.96)
LR Floor Q3	1.55 (1.07, 2.25)	1.50 (1.01, 2.22)	1.32 (0.82, 2.12)	1.51 (1.04, 2.19)	1.54 (1.06, 2.22)	1.38 (0.94, 2.03)	1.51 (1.04, 2.19)	1.23 (0.81, 1.87)
LR Floor Q4	1.51 (0.94, 2.44)	1.44 (0.89, 2.34)	1.91 (0.99, 3.67)	1.43 (0.88, 2.34)	1.50 (0.92, 2.45)	1.38 (0.83, 2.28)	1.48 (0.91, 2.40)	1.56 (0.88, 2.76)
LR Sofa Q2	1.27 (0.73, 2.19)	1.24 (0.72, 2.14)	0.92 (0.48, 1.74)	1.22 (0.70, 2.11)	1.27 (0.74, 2.18)	1.18 (0.69, 2.02)	1.24 (0.72, 2.12)	1.04 (0.60, 1.80)
LR Sofa Q3	1.26 (0.72, 2.21)	1.22 (0.69, 2.16)	1.27 (0.68, 2.36)	1.20 (0.68, 2.11)	1.27 (0.73, 2.23)	1.14 (0.65, 2.01)	1.22 (0.70, 2.12)	1.09 (0.63, 1.89)
LR Sofa Q4	2.81 (1.52, 5.21)	2.73 (1.45, 5.11)	2.57 (1.38, 4.77)	2.64 (1.43, 4.88)	2.87 (1.54, 5.35)	2.57 (1.39, 4.76)	2.71 (1.47, 4.97)	2.39 (1.41, 4.07)
House Mean Q2	0.99 (0.58, 1.69)	0.94 (0.55, 1.61)	0.96 (0.59, 1.56)	0.96 (0.56, 1.64)	1.00 (0.59, 1.68)	0.88 (0.51, 1.53)	0.95 (0.55, 1.63)	1.20 (0.66, 2.20)
House Mean Q3	1.98 (1.12, 3.50)	1.91 (1.08, 3.37)	1.62 (0.95, 2.78)	1.87 (1.06, 3.31)	1.94 (1.10, 3.42)	1.76 (0.98, 3.14)	1.91 (1.08, 3.37)	2.05 (1.25, 3.38)
House Mean Q4	1.59 (1.06, 2.38)	1.52 (1.01, 2.27)	2.20 (1.41, 3.43)	1.49 (0.99, 2.24)	1.57 (1.06, 2.33)	1.43 (0.94, 2.16)	1.53 (1.02, 2.30)	1.96 (1.24, 3.09)
House Max Q2	1.41 (0.77, 2.60)	1.38 (0.75, 2.54)	1.03 (0.61, 1.75)	1.36 (0.75, 2.48)	1.40 (0.77, 2.55)	1.28 (0.69, 2.39)	1.39 (0.76, 2.55)	1.40 (0.88, 2.22)
House Max Q3	1.96 (1.22, 3.17)	1.90 (1.19, 3.06)	2.78 (1.72, 4.47)	1.87 (1.16, 3.01)	1.91 (1.17, 3.13)	1.82 (1.12, 2.95)	1.93 (1.20, 3.13)	2.21 (1.33, 3.69)
House Max Q4	1.81 (1.18, 2.75)	1.74 (1.13, 2.68)	2.43 (1.41, 4.18)	1.69 (1.11, 2.56)	1.77 (1.16, 2.70)	1.67 (1.09, 2.55)	1.76 (1.16, 2.66)	2.09 (1.32, 3.31)
